# Senescent Cells: A Therapeutic Target in Cardiovascular Diseases

**DOI:** 10.3390/cells12091296

**Published:** 2023-05-02

**Authors:** Masayoshi Suda, Karl H. Paul, Tohru Minamino, Jordan D. Miller, Amir Lerman, Georgina M. Ellison-Hughes, Tamar Tchkonia, James L. Kirkland

**Affiliations:** 1 Department of Physiology and Biomedical Engineering, Mayo Clinic, 200 First St., S.W., Rochester, MN 55905, USA; 2 Department of Cardiovascular Biology and Medicine, Juntendo University Graduate School of Medicine, 3-1-3 Hongo, Bunkyo-ku, Tokyo 113-8421, Japan; 3 Department of Physiology and Pharmacology, Karolinska Institutet, Solnavägen 9, 171 65 Solna, Sweden; 4 Japan Agency for Medical Research and Development-Core Research for Evolutionary Medical Science and Technology (AMED-CREST), Japan Agency for Medical Research and Development, Tokyo 100-0004, Japan; 5 Division of Cardiovascular Surgery, Mayo Clinic College of Medicine, 200 First St., S.W., Rochester, MN 55905, USA; 6 Department of Cardiovascular Medicine, Mayo Clinic, 200 First St., S.W., Rochester, MN 55905, USA; 7 Centre for Human and Applied Physiological Sciences, School of Basic and Medical Biosciences, Faculty of Life Sciences & Medicine, Guy’s Campus, King’s College London, London SE1 1UL, UK; 8 Centre for Stem Cells and Regenerative Medicine, School of Basic and Medical Biosciences, Faculty of Life Sciences & Medicine, Guy’s Campus, King’s College London, London SE1 1UL, UK; 9 Division of General Internal Medicine, Department of Medicine, Mayo Clinic, 200 First St., S.W., Rochester, MN 55905, USA

**Keywords:** cellular senescence, senolytics, SASP, atherosclerosis, cardiovascular diseases

## Abstract

Senescent cell accumulation has been observed in age-associated diseases including cardiovascular diseases. Senescent cells lack proliferative capacity and secrete senescence-associated secretory phenotype (SASP) factors that may cause or worsen many cardiovascular diseases. Therapies targeting senescent cells, especially senolytic drugs that selectively induce senescent cell removal, have been shown to delay, prevent, alleviate, or treat multiple age-associated diseases in preclinical models. Some senolytic clinical trials have already been completed or are underway for a number of diseases and geriatric syndromes. Understanding how cellular senescence affects the various cell types in the cardiovascular system, such as endothelial cells, vascular smooth muscle cells, fibroblasts, immune cells, progenitor cells, and cardiomyocytes, is important to facilitate translation of senotherapeutics into clinical interventions. This review highlights: (1) the characteristics of senescent cells and their involvement in cardiovascular diseases, focusing on the aforementioned cardiovascular cell types, (2) evidence about senolytic drugs and other senotherapeutics, and (3) the future path and clinical potential of senotherapeutics for cardiovascular diseases.

## 1. Introduction

Age-related diseases, including cardiovascular diseases, are becoming more prevalent due to the increasing older population worldwide [1,2]. These diseases cause morbidity and mortality and contribute heavily to health care costs [3]. Consistent with this, epidemiological studies have shown that aging itself is a major predictor for many chronic diseases, including cardiovascular diseases [4]. The drug arsenal targeting the risk factors for cardiovascular diseases such as hypertension, diabetes, and hypercholesterolemia has grown exponentially, and the prevalence and mortality rates of cardiovascular disease have improved accordingly. However, even after strict lipid and hypertension control with statins, angiotensin-converting enzyme (ACE) inhibitors, and angiotensin receptor blockers (ARBs), cardiovascular event rates have not dropped precipitously. This may be because while early risk factor mitigation may be capable of attenuating the impact of fundamental aging mechanisms that particularly relate to cardiovascular disease, many of these approaches fail to effectively modulate “already established” fundamental mechanisms of aging, including senescent cell accumulation. Recognizing the importance of fundamental aging processes in cardiovascular disease development pertains to many potential new targets for pharmaceutical intervention, which may tip the scales to reduce the burden of cardiovascular diseases.

Senescent cell accumulation is a fundamental aging process. Accumulating in vivo and in vitro evidence indicates that cellular senescence contributes to chronic disorders such as cardiovascular diseases [5,6,7,8], type 2 diabetes and other age-related metabolic conditions [9,10,11,12,13,14,15,16,17], liver disease in association with hyperinsulinemia, metabolic syndrome, age-dependent steatosis [18,19,20], idiopathic pulmonary fibrosis and other lung disorders [21,22,23], kidney dysfunction in diabetic nephropathy and obesity [24,25], radiation-induced and age-related osteoporosis (as distinct from estrogen deficiency-related osteoporosis) [26,27,28,29], endometrial and uterine dysfunction [30], cognitive dysfunction, anxiety associated with obesity, dementias [31,32], and cancers and their complications [33]. Among these detrimental effects of senescent cell accumulation, complications in the cardiovascular system have been the subject of several recent studies. For example, senescent endothelial cells (ECs) exhibit functional abnormalities, such as decreased expression of endothelial nitric oxide (NO) synthase and increased expression of proinflammatory molecules, contributing to vascular inflammation [34,35,36]. Senescent ECs and other senescent cell types in the cardiovascular system share characteristics with other types of dysfunctional cells. However, there is difference between senescent cells and other dysfunctional cell types that potentially contribute to CVD: senescent cells are in a state of proliferative arrest, restricting repair of damaged tissues. These and other findings point to the importance of research into therapies directly targeting aging biology to alleviate age-associated diseases, reduce healthcare burden, and improve the quality of life for older individuals. Interventions known to enhance healthspan or extend lifespan in mice and other mammals, such as caloric restriction, have been shown to decrease senescent cell burden [37]. We therefore began to devise strategies for testing if selectively targeting and removing senescent cells in vivo can delay, prevent, alleviate, or treat disorders and diseases related to fundamental aging processes. Senolytic agents selectively eliminate those senescent cells that are tissue-damaging. Although the impact of senolytics is now backed by considerable evidence from preclinical studies and early clinical trials, a better understanding of cellular senescence in different types of cells, developing clinically useful markers of senescent cell abundance, and more data about the effects of these cells may speed the pace at which senolytics can be translated into clinical application.

## 2. Molecular Mechanisms of Cellular Senescence

Human cells have limited replicative potential, as discovered by Hayflick and Moorehead in 1961 [38]. They termed this loss of proliferative capacity “cellular senescence” and suggested that human aging can occur at the cellular level. Cellular senescence as initially discovered is related to telomeric dysfunction [39]. Telomeres are non-nucleosomal DNA-protein complexes with 5′-TTAGGGG-3′ repetitive sequences in human cells. They are located at both ends of chromosomes and protect and stabilize the genome and chromatin structure. In organisms that have linear DNA, telomeres are depleted with each cell division and restrict replicative potential, contributing to replicative cellular senescence [40]. Past a critical threshold, telomere shortening and telomere damage (even without shortening), are related to chronic DNA damage, with activation of cell cycle inhibitors, including p53/p21^CIP1/WAF1^ or p16^INK4a^/Rb, that induce cell cycle arrest in the G1 phase. Apart from telomeric dysfunction, DNA damage responses and subsequent cellular senescence can also be triggered by stimuli including oncogenes, radiation exposure, and chemotherapy [40]. Cellular senescence appears to be linked to a variety of senescence initiators, including DNA damage (by alkylating agents or radiation, causing accelerated telomere attrition or mutations), oncogenes (Ras, Myc, etc.), reactive oxygen species (ROS), reactive metabolites (ceramides, fatty acids, high glucose), mitogens, proteotoxic stress (protein aggregation, unfolded protein response, mammalian target of rapamycin (mTOR) activation), inflammation, damage-associated molecular pattern factors (DAMPs), pathogen-associated molecular profile factors (PAMPs), progenitor cell dysfunction, and epigenetic changes, among others [41,42]. These initiators might interact and amplify the detrimental effects of each other and senescent cells are also known to produce senescence initiators. An example is ROS, which causes DNA damage. Senescent cells formed due to this DNA damage also have increased ROS production, leading to a feed forward cycle. In the CVD setting, established risk factors such as angiotensin II (Ang II) [43], excessive insulin signaling [44], and hyperlipidemia [45] contribute to increased ROS, DNA damage, and increased senescent cell accumulation. Autophagy is critical for cellular homeostasis as well as cellular senescence and apoptosis. Cellular senescence and autophagy are commonly associated with detrimental processes including oxidative stress, DNA damage, telomeric dysfunction, and oncogenic stress. In addition, cellular senescence and autophagy share a common molecular mechanism: the mTOR pathway and lysosomal signaling. It is known that elevated levels of autophagy induce cell death, whereas inadequate autophagy can trigger cellular senescence and might contribute to CVDs [46].

Cellular senescence per se can contribute to tumor suppression. In response to DNA damage, cells enter cell cycle arrest, which suppresses uncontrolled proliferation of dysfunctional or cancerous cells. Cells that complete DNA repair can proliferate again. However, cells in which DNA repair has failed are eliminated by apoptosis. Those cells that escape such elimination become senescent and enter cell cycle arrest but are resistant to removal by apoptosis due to senescent cell anti-apoptotic pathways (SCAPs) [47]. Senescent cells can cause tissue dysfunction but remain viable and metabolically active. These cells can hinder tissue repair. As a result, tissues with high senescent cell burden can become dysfunctional. Furthermore, senescent cells can secrete pro-inflammatory, pro-fibrotic, and pro-apoptotic factors, such as interleukin-1 (IL-1), interleukin-6 (IL-6), tumor necrosis factor-alpha (TNF-α), and proteases such as plasminogen activator inhibitors 1 and 2 (PAI-1 & -2) or matrix metalloproteases (MMPs), elements of the senescence-associated secretory phenotype (SASP) [48,49,50,51]. The SASP can induce immune cell attraction, anchoring, and activation to remove senescent cells. As the immune system becomes dysfunctional with increasing age, senescent cells can avoid such removal. Senescent cells can also express “don’t eat me” signals such as the immune checkpoint programmed cell death protein 1 (PD-1), cytotoxic T-lymphocyte-associated protein 4 (CTLA-4), and major histocompatibility complex molecules (MHC class I). These markers of self-tolerance further hinder immune system clearance. In addition, senescent cells can release IL-17, which activates IL-23 and other factors within T lymphocyte subtypes that impede senescent cell removal. Therefore, senescent cells can accumulate in tissues, cause chronic inflammation, and contribute to a wide range of diseases.

## 3. Markers of Cellular Senescence

Markers that can be expressed by senescent cells include the cell cycle arrest proteins p53/p21^CIP1/WAF1^ and p16^INK4a^/Rb, DNA damage-related factors such as gamma-H2AX (γ-H2AX), p38 mitogen-activated protein kinase (p38MAPK) activity [52,53], telomere-associated foci (TAFs) [54,55], and senescence-associated β-galactosidase activity (SA-βgal) activity [56]. However, none of these markers are fully sensitive and specific. Indirect ways of assessing senescent cell burden include assays of SASP factors in blood, tissues, or cells. Examples of such SASP factors include interleukin-1α (IL-1α), IL-6, interleukin-8 (IL-8), IL-17, plasminogen activator inhibitors 1 and 2 (PAI-1, PAI-2), and profibrotic metalloproteases such as MMP-2, MMP-9, and MMP-12 [48]. Pregnancy-associated plasma protein A (PAPP-A), a metalloprotease that increases insulin-related growth factor (IGF) signaling by cleavage of IGF-binding protein 4, can be a SASP component [57,58]. Inhibition of PAPP-A by genetic modification was shown to increase lifespan in murine models [59,60,61]. However, SASP factors are not reliable as senescence markers if used individually because these molecules can be released by other cell types [62,63,64,65,66,67]. Another strategy for estimating senescent cell burden is to combine assays and measurements as a panel [68,69,70]. Among the first such blood composite score indicative of senescent cell burden and effect of senolytic drugs was examined in a study of senolytics administered to obese young adults with diabetes [71]. This composite panel of 10 blood SASP factors was significantly reduced following administration of senolytics compared to baseline and mirrored declines in senescent cell abundance in fat biopsies from these subjects. Many others have published or are developing composite scores. An example is the 125 gene-wide “SenMayo” panel, which uses genes identified from various age-related datasets in a transcriptome-wide approach for assaying tissue samples by whole-transcriptome and single-cell RNA-sequencing (scRNA-seq). This yields a senescence score that was reduced in bone samples after genetic clearance of senescent cells in mice as well as in human adipose tissue following pharmacological senescent cell clearance [72]. These data suggest that a combination of markers in a panel can be used to estimate senescent cell abundance across tissues and species.

With senolytic clinical trials underway, there is a need for biomarkers, “gerodiagnostics”, for following the therapeutic effects of these drugs. These should preferably be measurable non-invasively in blood or other body fluids such as urine or saliva. Recent studies have proposed quantifying p16 expression of CD3-positive T cells in peripheral blood as a relatively non-invasive way to assess senescent cell burden [73,74]. Post hoc analysis of the first clinical trial of senolytics revealed higher levels of the ‘geroprotective’ factor α-Klotho in urine after oral D+Q administration in each of 20 subjects with idiopathic pulmonary fibrosis [75]. In addition to other SASP factors, senescent cells can release small extracellular vesicles (EVs), including exosomes. Molecules in EVs obtained from the blood could be novel gerodiagnostic markers [76,77]. The microRNA (miRNA) expression and secretion profile of senescent cells has recently come into the spotlight [78,79]. These short, non-coding RNAs are involved in post-transcriptional regulation. They may represent a sensitive way to identify senescent cells.

The search for a single, common senescence marker for various senescent cell types has to date not been successful. Senescent cells often express different molecules depending on the initial trigger of senescence (e.g., oncogene-induced senescence [OIS] vs. therapy-induced senescence [TIS]), the cell type that become senescent, how long the cells have been senescent, and the tissue microenvironment). One approach to detect senescent cells is to classify them by senescence markers, cell type, and localization, focusing on one sub-group of senescent cells at a time. A recent example of this approach was in studies of adipose tissue senescence induced by a high-fat diet, where upregulation of p21^Cip1/Waf1^ but not p16^Ink4a^ was found [11,80]. Recent advances in single cell analysis, including single cell or nuclear RNA-seq, flow cytometry, or flow mass cytometry (CyTOF), can reveal information unavailable in bulk analysis of tissues. Cell subsets are already being classified using cell surface markers in the field of immunology. An example is the monocyte subset Ceacam1^+^Msr1^+^Ly6C^-^F4/80^-^Mac1^+^ [81] which has been shown to be critical for fibrosis. Recently, CD153^+^PD-1^+^CD4^+^ T cells were identified as senescence-associated T (SAT) cells [82] and a peptide vaccine targeting CD153 was successfully senolytic [83]. Classifying senescence in cardiovascular system cells, such as ECs, vascular smooth muscle cells (VSMCs), cardiomyocytes, immune cells, fibroblasts, and progenitor cells, will potentially be valuable. For example, expression of adhesion molecules attracting immune cells, such as intercellular adhesion molecule-1 (ICAM-1) and vascular cell adhesion molecule-1 (VCAM-1), has been found to increase in senescent endothelial cells. Although not as useful as global senescence markers, these could be used as cell-type specific senescence markers together with classical markers (Table 1). Ultimately, a goal of the field is to develop composite scores of analytes not only in body fluids, but potentially also including questionnaire data, physical findings, imaging, and wearable-device data. For the cardiovascular system, physiological indicators such as electrocardiogram, diastolic dysfunction measured by echocardiogram, endothelial function assayed by ankle brachial index (ABI) test, skin perfusion pressure measurement (SPP), flow dilation (FMD), near-infrared spectroscopy (NIRS), peripheral microvascular endothelial function (PMEF), and functional thrombus assays may also useful. Ideally, such a composite score might be able to estimate senescent cell burden, track fundamental aging mechanisms, predict disorders and diseases linked to these mechanisms, select the best anti-senescence interventions for each patient, and perhaps even be acceptable to regulatory agencies as primary outcomes of clinical trials. Developing such assays and composite scores across multiple clinical trials of geroscience interventions has been a priority of the Facility for Geroscience Analysis of the NIH Translational Geroscience Network (R33AG 61456).

## 4. Detrimental Effects of Certain Types of Senescent Cells in Cardiovascular Diseases

**Figure 1:**
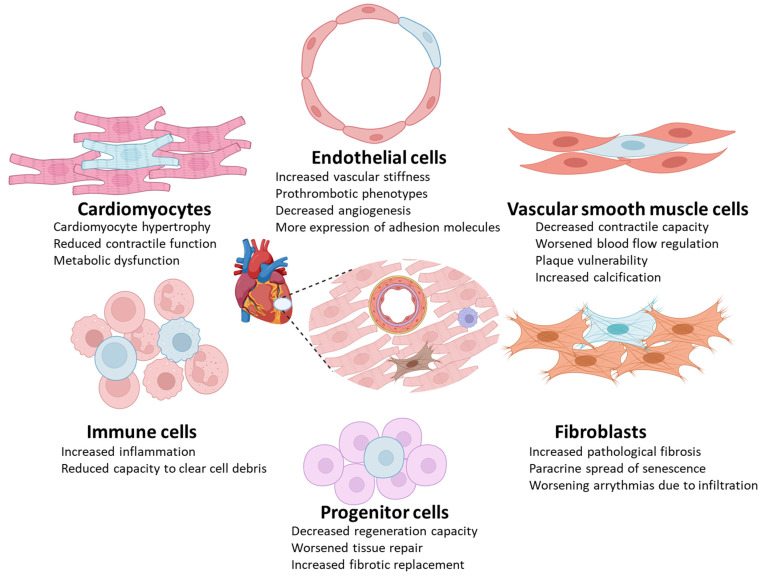
Consequences of senescent cell accumulation in cardiovascular diseases and the mechanisms behind disease development.

Accumulating evidence from in vitro and in vivo studies has revealed the molecular mechanisms behind senescent cell contribution to onset and progression of cardiovascular disorders. Here, we summarize the molecular patterns leading to cellular senescence and how various senescent cell types in the cardiovascular system can contribute to disease. (Figure 1, Table 2).

### 4.1. Endothelial Cells

Endothelial cells are constantly exposed to circulating blood and pathogenic stimuli and flow/shear stress that predispose them to damage and induce senescence. Consequently, senescent cell burden is high in well-vascularized tissues with many endothelial cells [84]. Endothelial cells are also one of the first cell types to manifest increasing effects of biological age [85]. PMEF as an index of vascular aging correlates better with advanced vascular aging as estimated by artificial intelligence (AI) analyses of ECGs than with actual age. This suggests that vascular aging as manifested by endothelial function is associated with accelerated physiological aging [86]. The hemodynamic environment in blood vessels is an important factor, evidenced by the fact that endothelial cells exposed to high shear stress have high cellular turnover that predisposes to replicative senescence [87,88]. Lifestyle-related metabolic conditions, such as dyslipidemia, are risk factors for cardiovascular diseases and promote endothelial senescence. Dyslipidemia propagates endothelial cell senescence due to increased oxidation of low-density-lipoproteins [45]. Type 2 diabetes may also cause endothelial cell senescence due to increases in circulating glucose and excessive insulin signaling [44]. Oxidized low-density-lipoproteins and insulin activate the phospho- inositol 3-kinase (PI3K)/RACα serine/threonine protein kinase (AKT1) signaling pathway, inhibiting forkhead box protein O(FOXO) 3A, resulting in reduced manganese superoxide dismutase (SOD) activity and a subsequent increase in reactive oxygen species (ROS). In vivo and in vitro studies have demonstrated that high glucose levels promote endothelial cell senescence by NADPH oxidase-, SOD-, and sirtuin- (SIRT) mediated ROS accumulation and increased pro-inflammatory SASP factor production [89,90]. ROS trigger the p53/p21^CIP1/WAF1^ DNA damage response pathway, inducing proliferative arrest and cellular senescence. Chemotherapeutic drugs such as doxorubicin are also known to increase mitochondrial ROS [91] and production of arterial SASP factors [92].

Senescent ECs exhibit functional abnormalities such as decreased expression of vasoprotective factors and increases in inflammatory cytokines and adhesion molecules. Senescent ECs also dysregulate blood flow and cause barrier dysfunction. They do not proliferate, impeding capacity to repair the blood vessel lumen. ECs produce the potent vasodilatory molecule nitric oxide (NO), which in addition to its vasodilatory effect mediated by VSMC relaxation, also reduces endothelial expression of adhesion molecules, improves barrier function, and prevents coagulation [93]. Furthermore, NO may have a protective effect against cellular senescence [94]. Consequences of senescence-driven EC dysfunction include reduced vascular dilation, partly due to increased p53 activity leading to decreased active endothelial nitric oxide synthase (eNOS) levels [95]. Inhibiting p53 activation in endothelium has been demonstrated to increase eNOS production [96]. Evidence exists that high glucose levels reduce telomerase activity, length, and phosphorylation of eNOS, thereby reducing NO production [97]. Persistent senescent endothelial cells can induce increases in Ang II [98] and endothelin 1 (ET1) levels [99], contributing to elevated blood pressure. Reduced NO synthesis and increased Ang II levels, along with studies of senescence markers, connect EC senescence to a common cardiovascular risk factor, hypertension. As the endothelium effectively is an endocrine tissue, senescent ECs may cause wide-ranging systemic consequences [100]. Reduced vasodilation due to senescent endothelial cell accumulation has also been associated with heart failure with preserved ejection fraction (HFpEF) [101]. Senescent endothelial cells exhibit increased production of ROS and certain SASP markers, such as intercellular adhesion molecule-1 (ICAM-1) [102,103], IL-1 [104], IL-6 [105], IL-8 [106], and MCP-1 [106], facilitating immune cell infiltration of tissues. Senescent ECs cause increased thrombosis risk and susceptibility to atherosclerosis due to increased plasminogen activator-1 (PAI-1) expression as well as reduced endothelial nitric oxide synthase (eNOS) [107]. Cyclo-oxygenase activity is altered in senescent ECs, as demonstrated by decreased prostaglandin I2 levels and increased thromboxane A2 production. The SASP factors considered above, including inflammatory proteins and growth factors, are recognized risk factors for atherosclerosis. Unsurprisingly, senescent ECs are found in high numbers in human atherosclerotic plaques [5]. Recent studies have identified endothelial cell senescence as a contributor to pulmonary hypertension. EC senescence seems to drive dysfunctional phenotypes through upregulation of several canonical SASP factors, such as IL-6, TNF-α, and PAI-1, leading to dysfunction in other vascular cells [108,109]. Angiogenesis is another aspect of endothelial function, protecting tissues against ischemia in ischemic heart disease and peripheral arterial diseases, primarily through vascular endothelial growth factor (VEGF) [110], fibroblast growth factor (FGF) [111], and hypoxia-inducible factor (HIF) 1α [112]. Senescent ECs decrease expression of these factors in vessels due to signaling through the p53-p21 senescence pathways, as evidenced by increased angiogenesis after this pathway is inhibited [96]. Angiogenesis may be associated with the development and vulnerability to atherosclerotic plaques. Further studies investigating this possibility are needed.

### 4.2. Vascular Smooth Muscle Cells

Vascular smooth muscle cells (VSMCs) are the main drivers of atherosclerosis, but senescent VSMCs appear not to be involved in initial plaque formation; rather, they may primarily lead to increased plaque size [113]. Accumulation of senescent VSMCs in atherosclerotic plaques is evidenced by reduced proliferation [114], larger and flatter cell morphology [115], increased expression of p16 ^INK4a^ and p21 ^CIP1/WAF1^ [116], and shortened or dysfunctional telomeres [116]. VSMC senescence can be caused by many factors, such as chronic inflammation, dysregulated local calcium metabolism, and increased oxidative stress. Conditions considered to be significant risk factors for atherosclerotic cardiovascular disease such as diabetes, hypertension, dyslipidemia, and smoking increase reactive oxygen species (ROS) in the vessel wall [117]. One significant source of ROS is nicotinamide dinucleotide phosphate (NADPH) oxidases (NOXs) [118]. Accelerated senescence of aortic VSMCSs, increased DNA damage, and a proinflammatory secretory profile were found in young mice with NOX4 overexpression, with the suggested mechanism being increased superoxide and hydrogen peroxide production [118]. ROS have also been tied to accelerated telomere depletion, which potentially contributes to the finding that VSMCs covering atherosclerotic plaques exhibit telomere shortening [116]. VSMCs are influenced by neural and endocrine signaling, and crucially, by paracrine mediators from ECs. The renin-angiotensin-aldosterone system (RAAS) is a regulator of VSMC activity, and high levels of Ang II are connected to VSMC senescence [43]. Inhibition of RAAS signaling may attenuate premature senescence and proinflammatory cytokine production caused by Ang II [43]. Other signals driving VSMC senescence are chronic exposure to high levels of the coagulation factor Xa [119,120], leading to chronic inflammation and stimulating p53 and insulin-like growth factor binding protein 5 (IGFBP-5) expression [121]. The monocyte chemotactic protein-1 and transforming growth factor-ß signaling pathways are additional players in VSMC senescence [122]. There are proteins that may delay cellular senescence, such as sirtuins [123]. In mice, sirtuin protein 6 (SIRT6) may counteract onset of senescence in VSMCs. Other beneficial effects of sirtuin activation may be increased atherosclerotic plaque stability, protection against telomeric damage, and reduced inflammatory cytokine production [124].

As VSMCs senesce, they can lead to increased production of proinflammatory proteins such as IL-1α, IL-1β, IL-6, IL-8, IL-18, and TNF-α [125]. These SASP factors and reduced anti-inflammatory protein expression drive chemotaxis of immune cells and increase endothelial cell adhesion molecule expression. Collagen production by senescent VSMCs and surrounding cells is consequently reduced, making atherosclerotic plaques more vulnerable to rupture [126,127,128]. Moreover, senescent VSMCs have increased production of elastase and other matrix-degrading proteases and add less collagen to the surrounding extracellular matrix (ECM) [129]. The contractile capacity of aged VSMCs is reduced [130] due to reduced expression of proteins involved in muscle contraction, such as α-smooth muscle actin (α-SMA), smooth muscle myosin heavy chain (SM-MHC), and calponin [131,132]. Ion channel types on the cellular membrane change: their numbers decrease and responses to mediators secreted by endothelial cells are diminished [131,132]. These changes may contribute to hypertension due to blood flow dysregulation and arterial stiffness. Senescent VSMCs may further contribute to this stiffness due to increased production of osteogenic mediators such as osteopontin (OPN), osteoprotegerin (OPG), runt-associated transcription factor 2 (Runx-2), bone morphogenetic protein 2 (BMP-2), and alkaline phosphatase (ALP) as a response to oxidative stress and inflammation [125,133]. Attenuators of this calcification include myostatin, an activator of the mTOR pathway that reduces osteogenetic factor expression [134]. Interestingly, no correlation was found between p21-expressing VSMCs and pulmonary hypertension, suggesting that senescent VSMCs may not be a primary driver of this disease [135].

### 4.3. Cardiomyocytes

As cardiomyocytes are terminally differentiated, post-mitotic cells in adult humans (with a yearly renewal capacity of less than 1% [136]), senescence in cardiomyocytes is challenging to define precisely compared to proliferative cells. Evidence from animals and humans has indicated that post-mitotic cardiomyocyte senescence is mediated by length-independent telomeric damage [54,137]. In mice, it has been reported that cardiomyocyte size, ROS production, and p53 or p16^Ink4a^ expression increases with chronological age together with telomere attrition [54,138]. These findings are signs of cellular senescence also encountered in senescent normally proliferating cell types. In addition to biological aging, senescent cardiomyocytes may be implicated in multiple pathologies, including dysfunctional remodeling after myocardial infarction [139] and hypertrophic cardiomyopathy. In heart failure due to sustained pressure overload, p53-expressing cells were present in increased numbers. Senescent cell accumulation was shown to inhibit Hypoxia-Inducible Factor (HIF)-1 activity and impair cardiac angiogenesis and systolic function [112]. Cardiomyocyte remodeling is initiated by p53-independent mitochondrial activation and is characterized by hypertrophy, but continuous stimuli from volume overload led to p53-dependent mitochondrial inhibition, morphological elongation, and heart failure [140]. Cardiotoxic drugs such as doxorubicin can induce premature senescence in cardiomyocytes as evidenced by increases in p16^Ink4a^ and p53/p21^Cip1/Waf1^ expression, increased SA-ßgal signaling, decreased cardiac troponin I phosphorylation, and decreased telomerase activity [141,142], potentially contributing to doxorubicin-induced cardiomyopathy. Paracrine mediators from ECs, fibroblasts, and immune cells are possibly also drivers of cardiomyocyte senescence. ECs promote cardiomyocyte maturation during infancy through growth factors such as platelet-derived growth factor (PDGF). With increasing age, senescent ECs instead drive inflammation and senescence in cardiomyocytes through release of factors such as tumor growth factor-β (TGFß) and IL-6. Decreased NO production by senescent ECs directly exacerbated cardiomyocyte contractility by disrupting the fine regulation of excitation–contraction coupling, dysregulating autonomic signaling, and impacting mitochondrial respiration in addition to vascular-dependent aspects such as increasing vascular stiffness, inflammation, thrombosis, and impairment of angiogenesis [143]. Consistent with this, endothelial senescence has been demonstrated to play a key role in heart failure. Senescent fibroblasts also secrete these markers, along with TNF-α and IGF-1. Immune cells such as macrophages may drive senescence by producing factors such as IL-1ß. SASP factors from adipose tissue are also involved in cardiomyocyte aging and heart failure [144].

Senescent cardiomyocytes produce SASP factors, including proinflammatory cytokines and chemokines, growth modulators, angiogenetic factors, and matrix metalloproteinases (MMPs) [145]. Examples include CCN family member 1 (CCN1), interleukins (IL1α, IL1β, and IL6), TNF-α, MCP1, endothelin 3 (Edn3), TGFβ, and growth and differentiation factor 15 (GDF15) [54,146]. These factors can contribute to cardiac remodeling and dysfunction. As cardiomyocytes consume more energy than most other types of cells, well-functioning cellular metabolism is essential for homeostasis. Dysfunctional metabolism may contribute to cardiomyocyte senescence during aging and the development of various diseases. When cardiomyocytes become senescent, p53 upregulation shifts the cell toward glucose metabolism. This increases activation of the insulin growth factor receptor, promoting senescence and increasing the release of SASP factors. Age-related declines in mitochondrial enzyme levels may also cause further toxicity due to metabolite buildup. For example, carnitine palmitoyltransferase 1 (CPT1), one of the rate-limiting enzymes in the fatty acid oxidation and glucose oxidation pathways, has been shown to be decreased in cardiomyocytes of aging rats [147]. In addition, expression of other regulators of fat metabolism, such as peroxisome proliferator-activated receptor α (PPARα) and PGC-1α, is also reduced with age [148]. As these enzymes decrease with age, consequent intracellular lipid buildup may induce senescence. Core metabolism-regulating enzymes such as AMP-activated protein kinase (AMPK), NAD^+^-dependent sirtuins, FOXOs, and mammalian target of rapamycin (mTOR) play prominent roles in driving or inhibiting cardiomyocyte senescence. AMPK activation is reduced in aged myocardial tissues, and activation of AMPK improves mitochondrial dynamics, reduces ER stress, improves the function of cardiomyocytes, and represses cardiomyocyte senescence [149,150,151]. Sirtuins are NAD+-dependent cell metabolism and senescence regulators, and contractile dysfunction is linked to NAD+ depletion [152]. Increased mTOR activity, on the other hand, has been linked to pathologic cardiomyocyte hypertrophy.

### 4.4. Immune Cells

Immune cells play a prominent role in CVDs, as demonstrated by a report showing that atherosclerotic plaques with high macrophage content were more vulnerable to rupture [153,154,155]. The detrimental effects of immune cells may partly be due to SASP secretion that may increase inflammatory cell migration and cause local damage. For example, the SASP directly drives inflammation through IL-1α translocation to the cell surface, which activates neighboring VSMCs, ECs, and macrophages, causing the spread of inflammation, and promotes atherosclerosis through secondary proinflammatory cytokines [122].

Myocardial cellular composition includes resident immune cells such as subsets of leukocytes encountered in the healthy heart. In a recent study, flow cytometry of cardiac tissue showed approximately 10^3^ leukocytes per milligram of tissue in the steady state and 3380 ± 1279 CD45^+^ cells per cubic millimeter of tissue. Three-dimensional reconstructions of immunohistochemistry images detected CD45^+^ cells even within the healthy myocardium. Interestingly, cardiac muscle contained 12 times more leukocytes per milligram of tissue compared to skeletal muscle. Of all the leukocytes found in the healthy heart, only ∼13% were in direct contact with the bloodstream. Fundamental changes in cardiac leukocyte composition occur over time, affecting macrophages and T cells. The resident cardiac macrophage population (primarily CD206^+^ cells) significantly diminishes with aging, being replaced by granulocytes. Despite the reduction in macrophage numbers, aging does not affect the ratio of the F4/80^+^CD206^+^ and F4/80^+^CD206^−^ subsets. Still, a slight increase in C-C motif chemokine receptor 2-(CCR2)-expressing macrophages was observed in the hearts of 12- to 15-month-old animals, suggesting possible macrophage replenishment [156]. Cardiac macrophages have been reported to improve electrical conduction in the atrioventricular node, evidenced by cells being fused together with elongated macrophages expressing connexin-43 in the adult heart. Using the Cd11bDTR mouse model, macrophage ablation was shown to cause progressive atrioventricular blockage. These findings elucidate the role of macrophages in normal and aberrant cardiac conduction [157]. Macrophages lodge within the healthy myocardium phagocytose cell components, including mitochondria, of damaged cardiomyocytes. Immune system aging may increase formally senescent immune cell burden, potentially contributing to morbidity and mortality. After myocardial infarction, cellular debris from dead cardiac cells is cleared by neutrophil and macrophage phagocytosis. Depletion of cardiac macrophages results in defective elimination of mitochondria from myocardial tissue, inflammasome activation, impaired autophagy, abnormal mitochondrial accumulation in cardiomyocytes, metabolic alterations, and ventricular dysfunction [158]. With increasing age, cardiac macrophages appear to become senescent-like, starting to secrete damaging matrix metalloproteinases (MMPs) and CCL2, both shown to drive cardiomyocyte hypertrophy. The pathological effects of immunosenescence on CVDs can be further exacerbated by increased levels of osteopontin (OPN) and/or visceral obesity.

A recent study demonstrated that macrophages are the most common p16^Ink4a^/SA-βgal-positive cells accumulating in aging mice [159]. In the CVD setting, leukocytes with short telomeres, a sign of senescence, have been found in atherosclerotic coronary arteries. Senescent-like macrophages appear to have increased SA-β-Gal activity and p53 and p16 ^INK4a^ expression, display impaired cholesterol efflux, and exacerbate atherosclerosis [160,161]. Foamed macrophages exhibiting senescence markers may possibly secrete inflammatory cytokines, chemokines, and metalloproteinases in atherosclerotic plaques [162]. There are three distinct immune cell types in atherosclerotic plaques, each with a different morphology when analyzed by transmission electron microscopy (TEM). These three are the elongated, vacuolated cells located in the endothelial layer, spindly foam cells with histological properties of VSMCs, and large foamy cells resembling lipid-loaded macrophages producing X-galactosidase (X-Gal) crystals [162]. Oxidized low-density lipoprotein (LDL) inhibits macrophage proliferation and migration, induces cellular senescence, and promotes the secretion of inflammatory factors such as TNF-α, monocyte chemoattractant protein-1(MCP-1), and IL-1β, possibly establishing a positive feedback loop [163].

Besides macrophages, senescent-like T cells may also be involved in the pathogenesis of chronic inflammatory diseases, including vascular diseases [82]. In T cells, oxidative stress reduces telomerase activity, causing a T cell senescent-like state and creating proinflammatory phenotypes within plaques [164,165,166]. Senescent-like T cells with the CD8^+^CD57^+^CD27^−^CD28^null^ phenotype produce large amounts of IFN-γ and TNF-α and may promote inflammation in atherosclerotic disease [167]. Furthermore, telomere shortening in T cells has been observed in patients with atherosclerosis. Terminal restriction fragment (TRF) analysis has indicated that the mean length of the TRF in leukocytes of coronary artery disease (CAD) patients is shorter than in controls with no family history of CAD [168]. It is still unclear whether senescent-like T cell accumulation is the cause or the result of atherosclerosis; however, senescent T cells have been implicated in damaging VSMCs and ECs by producing perforin and granzymes, which may drive atherosclerosis [126]. Senescent-like T cells also secrete IFN-γ, which activates macrophages and increases metalloproteinase production [169]. The resulting ECM destruction may again play a part in atherosclerosis [46]. During aging, T cells accumulate potentially inflammatory cholesterol. The cholesterol efflux pathways behind this accumulation suppress T cell apoptosis and cause a senescent-like state, contributing to atherosclerosis in middle-aged mice [170]. Senescent T cells have been reported to drive hypertension. A higher frequency of CD57^+^CD28^−^CD8^+^ T cells and increased expression of CXCL11 has been noted in patients with hypertension compared to healthy controls, suggesting that immunosenescent cytotoxic CD8^+^ T cells are linked to hypertension [171]. Senescent CD4^+^ T cells producing interferon-gamma (IFN- γ) are found in high numbers in patients with acute coronary syndromes and may contribute to decline in myocardial function. Senescent CD8^+^ T cells have been connected to increased mortality six months after suffering a myocardial infarction [172]. In HIV-positive women, they were associated with carotid artery disease, and in CMV-seropositive patients, their numbers correlated with worsening left ventricular function [173]. IFN-γ-producing CD28^null^ CD4^+^ T cells were shown to accumulate in lymph nodes draining the heart of aged mice, and implanting these cells to young mice caused inflammation [156,174]. It has also been speculated that senescent CD4^+^ T cells might infiltrate the heart and promote inflammation and an increased stress response, causing heart failure [175]. Senescent CD57^+^CD8^+^ T cells have been observed in patients with acute myocardial infarction (MI) in higher concentrations than controls, and their numbers correlate with post-MI cardiovascular mortality [172]. Perhaps senescent cell IFNγ-driven IL-17 secretion may alter IL-23 levels and impact T lymphocyte subsets and contribute to post-MI dysfunction.

### 4.5. Progenitor Cells

Endothelial progenitor cells (EPCs) from the bone marrow can participate in postnatal neovascularization and vascular repair [176,177,178]. Declines in EPC function, proliferation, and telomere length are potentially detrimental to vascular EC function and contribute to reduced neovascularization and atherosclerosis [179,180]. In vitro, senescent EPCs form fewer capillaries and grow more slowly than non-senescent controls [181]. Senescent EPCs may impede vascular healing and worsen age-related vascular diseases.

Cardiac progenitor cells (CPC’s) can differentiate into cardiomyocytes, VSMCs, and ECs, and their myogenic potential is especially important since differentiated cardiomyocytes have low proliferative capacity. One characteristic of CPCs is expression of the protein c-kit; in the adult heart, only approximately 2% of the cells express this protein. Senescent CPCs accumulate in the heart right atrial appendage [7] and most CPCs in human hearts become senescent in old age [7]. Patients with cardiac diseases, particularly ischemic heart diseases, have damaged cardiomyocytes and may require myocardial regeneration from progenitor cells. The increasingly senescent CPCs may not be able to maintain homeostasis, repair damage, or regenerate after injury [182,183,184]. In chronic heart failure, senescent CPCs may hinder myocardial ability to regenerate and cause further fibrotic remodeling.

Senescent CPCs can produce proinflammatory and profibrotic SASP components such as IL-1ß, IL-6, IL-8, PAI-1, and MMP-3 [51]. These paracrine mediators spread senescence, as shown by conditioned media from senescent CPCs causing an increase in senescence markers in a non-senescent CPC population [7].

Cardiac tissue obtained from nonaged (50- to 64-year-old) patients with type 2 diabetes mellitus (T2DM) and without DM (NDM) and post-infarct cardiomyopathy had higher ROS production in T2DM, which was associated with an increased number of senescent/dysfunctional T2DM-human CPCs with reduced proliferation, clonogenesis/spherogenesis, and myogenic differentiation versus NDM-CPCs in vitro. Moreover, T2DM-CPCs expressed a pathologic SASP [185].

Further studies are needed to more fully elucidate the characteristics of senescent EPCs and CPCs and their role in CVD development. As of today, consensus about methods for identifying these cells and their function has not been reached. As discussed above, several groups have independently reported that senolytic administration improves CPC function and have suggested that progenitor cell senescence may become a target for future CVD interventions.

### 4.6. Fibroblasts

Cellular senescence was originally discovered by L. Hayflick et al. using fibroblasts [38]. In the cardiovascular system, fibroblasts may be the most abundant cell type. Increased biological age has been linked to fibroblast senescence, evidenced by fibroblasts containing X-Gal crystals in the pericardium. Recent studies have reported that senescent biomarkers, including p16^Ink4a^ and p21^Cip1/Waf1^, were increased in post-myocardial infarction mouse hearts, and costaining of α-SMA with p53 or p16 supports the possibility that senescent myofibroblast numbers are increased in infarct-border regions [186,187]. Senescent cardiac fibroblast accumulation was also noted in murine models of overload-induced cardiac hypertrophy. Senescence markers such as p16^Ink4a^, p21^Cip1/Waf1^, and SA-ßgal were higher in cardiac fibroblasts (CF), up to 20 times compared to sham models. These senescent cells accumulated within fibrotic areas [188]. Induced cardiac hypertrophy in transgenic mice with high ß1-adenoreceptor expression also increased the number of CFs expressing these three senescence markers. In heart biopsies from patients with idiopathic cardiomyopathy, there were significant increases in p16^INK4a^, p21^CIP1/WAF1^, and SA-ßgal positive cell populations. Another group found that heart tissue from patients undergoing ablation for atrial fibrillation (AF) exhibited increases in senescence markers co-localized with vimentin and α-SMA. Senescent fibroblasts were found to accumulate in the adventitial layer of blood vessels in the lungs of a pulmonary hypertension mouse model [189,190]. Another recently discovered marker of CF senescence is osteopontin (OPN) from peripheral adipose tissue, which potentially contributes to cardiac aging [191].

When activated by injury, resident cardiac fibroblasts (CFs) may transition into being myofibroblasts with α-smooth muscle actin expression, possibly helping to attenuate injury due to increased production of extracellular matrix (ECM) components. During and after repair, these myofibroblasts may mature into matrifibroblasts or return to their initial state. With continuous stress, CFs can undergo apoptosis or develop a senescent-like state with increased cell cycle-arresting proteins and SASP expression. Senescent CFs and myofibroblasts have been suggested to drive pathologic fibrosis and can be identified through their increases in platelet-derived growth factor, vimentin, and α-smooth muscle actin co-localized with senescence markers. Senescent CFs may contribute to cardiomyocyte senescence through paracrine signals and extracellular matrix modulation (ECM) [192]. Senescent fibroblasts also secrete IL-33, which attenuates cardiomyocyte senescence after hypoxic injury [193]. Fibroblasts normally express integrins, crucial for immune cell adhesion and surveillance and, through paracrine signaling to the ECM and the actin cytoskeleton, may contribute to ECM homeostasis [194]. Senescent fibroblasts and myofibroblasts are abundant within fibrotic areas and are involved in fibrotic myocardial pathologies. Interestingly, inducing senescence prematurely by CCN-1 decreases fibrosis in murine models [188]. This is in line with another finding demonstrating that a transient rise in the number of senescent fibroblasts reduces the fibrotic response after cardiac injury [195]. This may be due to senescent cells suppressing non-senescent fibroblast proliferation. Another explanation may be that senescent fibroblasts cannot proliferate and therefore cannot enlarge fibrotic areas. Oxidative stress may also be a contributor to AF development [196] and SASP factor release from senescent fibroblasts may worsen this condition.

## 5. Therapies Targeting Senescent Cells

**Figure 2:**
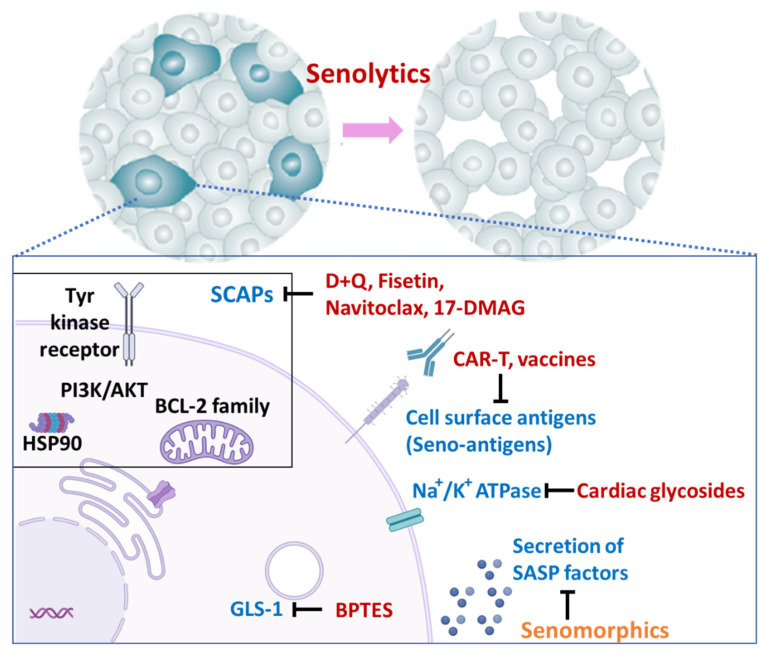
Targets of senolytics and senomorphics.

The senescent cells that accumulate with increasing age are damaged, dysfunctional cells without the proliferating capacity needed to repair tissue. These cells can promote chronic inflammation by producing SASP factors. The geroscience hypothesis is that targeting “hallmarks of aging”, such as cellular senescence, may delay, prevent, alleviate, or treat multiple chronic diseases [197]. Furthermore, since cellular senescence and other underlying causes of these age-associated chronic diseases (the hallmarks of aging) are interlinked with each other, intervening against any one of them may affect most or all of the other aging processes, as expressed in “The Unitary Theory of Fundamental Aging Mechanisms”, built on the Geroscience Hypothesis [197]. This theory is strengthened by the observation that about 80 percent of older adults have at least one chronic disease and 68 percent have at least two [198]. In addition to these diseases, multiple physical and mental health conditions, the geriatric syndromes, are associated with advanced age, including frailty, sarcopenia, cognitive impairment, and urinary incontinence. Advanced biological age is also associated with loss of physical resilience, such as against infection [199,200], injury, or surgery or dampened vaccination responses. Many or most of these syndromes might be alleviated by targeting the hallmarks of aging [201]. Novel strategies, such as senolytic agents (Figure 2), may therefore allow targeting aging mechanisms that contribute to causing multiple age-associated diseases. Studies are needed to test if senescence-targeting drugs added to existing disease-specific treatment regimens are effective in a potentially more than additive manner.

### 5.1. Elimination of Senescent Cells (Senolytics)

We tested if senescent cells with DNA damage and a pro-apoptotic SASP avoid undergoing apoptosis due to activation of senescent cell anti-apoptotic pathways (SCAPs) [47]. Bioinformatics analyses of proteomic data from different types of senescent and non-senescent human cells led to identification of such SCAPs. Key SCAP nodes were then inhibited using small interfering RNAs (siRNAs). This resulted in the 30 to 70% of senescent cells that are pro-apoptotic and tissue-damaging being eliminated through apoptosis. Different types of human pro-apoptotic senescent cells depend on different SCAPs for survival. Among others, these pro-survival signals in different senescent cell types include ephrin-(EFNB1 or 3) dependent SRC kinases, the phosphatidylinositol-4,5-bisphosphate 3-kinase delta catalytic subunit (PI3KCD), cyclin-dependent kinase inhibitor 1A (CDKN1A; p21^CIP1/WAF1^), BCL-xL, mitochondrial pathways, and plasminogen-activated inhibitor-2 (PAI-2) [42,202,203,204]. Next, bioinformatics approaches were used to search for small molecules that target these SCAPs. The SRC kinase inhibitor dasatinib (D) blocks SRC kinase/EFNB1/3-dependent suppression of apoptosis [205,206]. D, which has been approved by the FDA for use in humans since 2006 for treating hematologic cancers, eliminated the 30–70% of senescent human fat cell progenitors (MSCs) that are pro-apoptotic [47]. Quercetin (Q), a natural flavonol that interferes with PI3K and other SCAP pathways, induced death of tissue-damaging senescent ECs [47]. Combining dasatinib and quercetin (D+Q) reduced senescent cell burden in chronologically aged mice [47]. Mouse models have also been developed permitting elimination of some types of senescent cells, e.g., *INK-ATTAC* mice, from which highly *p16^Ink4a^*-expressing cells can be targeted for apoptosis using an agent, AP20187, that does not target cells without high expression of *p16^Ink4a^* [207]. However, it should be noted that since senolytics are based on targeting that subset of senescent cells with a pro-apoptotic SASP, while in *INK-ATTAC* mice it is those cells with high *p16^Ink4a^* expression that are targeted, the effects of senolytics could differ from those in *INK-ATTAC* mice treated with AP20187.

Transplanting small numbers of senescent cells into middle-aged mice is sufficient to cause frailty, physical dysfunction, and premature onset of most or all of the diseases found in older non-transplanted mice [208]. It was also shown that transplanting senescent cells can cause some of the recipients’ own cells to undergo senescence, even at a distance. Hence, senescence can spread not only by paracrine means, but also distantly in an “endocrine” fashion. Additionally, intermittently eliminating senescent cells alleviated physical dysfunction after senescent cell transplantation, all pointing toward a causal role of senescent cells in aging phenotypes and diseases [208]. These findings support the potential for targeting senescent cells as a therapeutic strategy to delay, prevent, alleviate, or treat multiple age-related pathologies [209]. Based on the *BCL-xL* siRNA studies in the first article about senolytics, two groups subsequently found that the BCL-xL/BCL-2/BCL-w inhibitor, navitoclax (ABT263), also exhibits senolytic activity [210,211]. Many more senolytic molecules were subsequently identified using the original hypothesis-based drug discovery approach and later by high-throughput screening. Examples include the specific BCL-xL inhibitors A1331852 and A1155463 [212], the flavonol fisetin [212,213], piperlongumine [203], procyanidin C1 [214], FOXO4-related peptide [215], and several dozen other small molecules [197]. Tissue-damaging senescent cells can take from 1 to 6 weeks to reaccumulate after senolytic administration in vitro. Hence, senolytics can be administered once or, if there is continued generation of senescent cells (e.g., sustained high-fat feeding), intermittently, once every couple of weeks or once a month. In mouse models, this “hit and run” approach appears to be effective [10,197,216]. Administering senolytics with short elimination half-lives may also reduce off-target or side effects compared to drugs that are administered continuously or drugs that have long half-lives, since intermittently administered senolytics have less opportunity to exert sustained effects on off-target receptors or enzymes. Potentially, this may also result in less benefit in vivo, as many senolytic compounds have been shown to exhibit effects that are possibly independent from their senolytic properties when administered continuously (e.g., flavonols can reduce ROS).

A new senolytic drug of interest is digoxin, a cardiac glycoside that has been used for many years for cardiac disease. Transplanted senescent cells are eliminated by digoxin administration in mouse models [217,218]. Repurposing existing drugs such as digoxin can hasten clinical translation. The results of clinical trials showing that chronic digoxin treatment reduced the rate of hospitalization of patients with heart failure but did not reduce overall mortality are well known [219]. Digoxin has a narrow safety window, especially in the elderly and patients with impaired renal function, and side effects such as bradycardia and lethal arrhythmia are often observed in long-term use. Senolytic therapy with digoxin, similarly to many other senolytic regimens, could be administered in an intermittent, hit-and-run fashion that may lead to better clinical outcomes. Glutaminase 1 (GLS1) is an amidohydrolase upregulated by a kidney-type glutaminase (KGA) that neutralizes the intracellular acidosis caused by lysosomal damage in senescent cells. Treatment with its inhibitor, BPTES [bis-2-(5-phenyl- acetamido-1,3,4-thiadiazol-2-yl) ethyl sulfide], appears to reduce senescent cell accumulation in obese and atherosclerotic mice [220]. As senescent cells sometimes have elevated activity of lysosomal β-galactosidase, galactose-modified cytotoxic prodrugs might be senolytic. Treatment with a galactose-modified duocarmycin (GMD) reduced senescent cell burden in adamantinomatous craniopharyngioma (ACP) model mice [221]. Progress has also been made in developing anti-senescent cell antibodies and vaccines. Screening of repurposed drugs and natural products and development of new molecules targeting senescent cells is ongoing. Immunotherapies developed as anti-cancer treatments, such as chimeric antigen receptor T cell (CAR-T) cells, cytotoxic T lymphocyte (CTL) therapy, natural killer (NK) cell therapy, and dendritic cell (DC) therapy, which are already in clinical application could be repurposed as senolytics by targeting senescent cell-specific antigens (seno-antigens). A potential advantage of such therapies is that, once a target molecule is identified, specific treatments can be developed even if the physiological function of the molecule is not understood or effective inhibitors have not been discovered. This approach could reduce off-target effects. GPNMB (glycoprotein nonmetastatic melanoma protein B) is a molecule that acts to maintain lysosomal homeostasis in senescent cells and was identified as a senescence marker by comparing young cells with replicative senescent cells [222]. GPNMB is upregulated in the aorta and adipose tissues of aged and obese mice, as well as in the aortae of an atherosclerosis mouse model. GPNMB expression is high in patients with atherosclerotic diseases. Interestingly, a senolytic vaccine targeting GPNMB alleviated diabetes and atherosclerosis-related complications in mouse models. This vaccine also improved physical function in aged mice and increased lifespan in progeroid mice [223]. Another senolytic vaccine targeting CD153, which has been reported as a marker of obesity-related T cells in adipose tissue, reduced accumulation of senescent T cells [83]. Glucose tolerance and insulin resistance were also improved. Both vaccine therapies were effective for several months. β2 microglobulin (B2M) is a component of the MHC class I molecules expressed on the cell surface and levels of this protein increase progressively with age in the brain and skin of mice. An antibody-drug conjugate (ADC) against B2M clears senescent cells through releasing duocarmycin near senescent cells [224]. Urokinase-type plasminogen activator receptor (uPAR) was identified by RNA sequencing as an antigen expressed on the cell surface of some types of senescent cells. CAR-T therapy has been tested against this antigen for senescent cell elimination. uPAR-specific CAR-T cells aggregated around uPAR-positive senescent cells and eliminated them, alleviating non-alcoholic steatohepatitis and drug-induced liver fibrosis in mouse models [225].

### 5.2. Other Strategies

Adipocyte-specific or EC-specific high-fat fed p53 knockout mice have improved metabolic function [43,95,226]. p53 antagonists are available [227]; however, systemically reducing the expression of the p16^Ink4a^ or p53-p21^Cip1/Waf1^ pathways would likely increase risk of cancer [228,229,230,231], because inhibiting the ability to form senescent cells appears to allow DNA-damaged cells to continue to proliferate. Even a small number of such cells could cause tumor formation. Hence, inhibiting the capacity to form senescent cells may therefore not be viable because senescence is a mechanism for preventing tumorigenesis.

Another possible strategy is to reprogram cells using the Yamanaka factors, Oct4, Sox2, Klf4, and c-Myc (OSKM), a discovery for which the Nobel prize was awarded in 2012 [232]. This approach allows cells, potentially including senescent cells, to be brought into a state akin to pluripotency [233,234,235]. In vitro experiments have indicated lengthening of telomeres and resetting of the gene expression profile to a “younger state” in cells from centenarians and patients with Hutchinson–Gilford progeria syndrome (HGPS). Interestingly, Yamanaka factor activation increases senescent cell formation, and a high senescent cell burden at the start of OSKM treatment has been linked to greater impact on cellular phenotypes. This increased senescent cell formation seems to require *Ink4a*/*Arf* locus activity. The same pathway is involved in IL-6 production and makes cells more receptive to OSKM factors [236]. A problem related to cellular reprogramming is the potential development of cancers such as teratomas and teratocarcinomas, since reprogramming may allow cancer-harboring senescent cells to reacquire proliferative potential. Attempts are being made to such circumvent cancer development [237,238,239,240]. This area is new, and it remains to be seen if reprogramming can be made safe and if causing cancers can be avoided.

SASP inhibitors, “senomorphic” drugs, do not eliminate senescent cells directly(Figure 2). They act by modifying aspects of the senescent cell secretome that cause chronic inflammation and tissue destruction. Some of these drugs act by targeting the transcription factor NF-κB [241,242], Janus kinases, or STAT signaling pathways. Other targets are the mTOR pathway, p38 mitogen-activated kinase (MAPK) and related kinases, mitochondrial complex 1-related or 4-related molecules, heat shock protein 90 (HSP90), and NAD^+^/NADH metabolism [243]. The effectiveness of senomorphic drugs was indicated in *p21^Cip1/Waf1^-Cre* mice in which removal of those cells highly expressing *p21^Cip1/Waf1^* can be induced. Inactivating the NF-κB pathway in cells with high *p21^Cip1/Waf1^* expression yielded the same results as removing the cells, and both methods attenuated insulin resistance in obese mice [11,80]. There are several FDA-approved medications that have senomorphic effects. For example, metformin, an established anti-diabetes medication, improves cardiovascular diseases [244] and cognitive dysfunction [245], attenuates cancer formation and resistance [246,247], and might increase lifespan [248]. Rapamycin, used clinically as an immunosuppressant, improved heart failure [249], cognitive decline [250], immune dysfunction [251], frailty [252], and possibly lifespan [253]. Ruxolitinib, in clinical use for various disorders (e.g., polycythemia rubra vera, myelofibrosis, and graft vs. host disease) [106] alleviated age-related adipose tissue dysfunction [15] and frailty [254]. After these encouraging findings, the American Federation for Aging Research (AFAR) and others are planning the TAME (Targeting Aging with Metformin) trial. This will test if metformin delays appearance of a second age-related disease in patients who already have an age-related condition. As it will not enroll patients with diabetes, the trial will also test the geroscience hypothesis [255,256]. A challenge in using certain senomorphic drugs, however, is modulating potential off-target effects, such as suppressed inflammation in the case of some diseases or tissue repair. Since, unlike senolytics, senomorphic drugs do not directly eliminate the senescent cells that are the cause of tissue-damaging SASP factor release, continuous treatment is potentially more necessary than is the case with senolytics.

## 6. Cardiovascular Diseases That Senotherapeutics May Potentially Alleviate

Several preclinical studies have tested senotherapies, including senolytics for cardiovascular dysfunction and diseases (Table 3). For example, Sunderland et al. showed that co-culture of human senescent cells with human iPSC-derived cardiomyocytes and endothelial cells leads to decreased survival and cell cycle activity [257]. Moreover, the endothelial cells exhibited impaired tube formation and migration. D+Q, by eliminating senescent cells, improved human iPSC-derived cardiomyocyte survival and DNA synthesis. D+Q also improved human endothelial cell survival, migration, and tube formation. The mechanism of action appears to involve abrogation of SASP factors by D+Q treatment, especially IL-6 and IL-8. A connection between senescence and fibrosis involving fibroblasts and cardiomyocytes has been made. In vitro, senescent fibroblasts can secrete profibrotic enzymes [23]. The first clinical trial in which the benefit of senolytics was reported in humans was in patients with idiopathic pulmonary fibrosis (IPF) [258]. Cardiac fibrosis, in which contractile heart muscle is replaced with connective tissue, is frequently seen in aged hearts. D+Q administration improves cardiac function in aged mice by attenuating senescence in post-mitotic cardiomyocytes [47,54]. In high-fat diet-treated mice and rats used as diabetic cardiomyopathy models, Q reduced cardiac fibrosis, normalized heart size, and attenuated cardiac systolic dysfunction [8,259]. Navitoclax also decreased cardiac fibrosis, hypertrophy, inflammation, and cardiac dysfunction in mice with doxorubicin-induced heart failure, a chemotherapy-induced cardiomyopathy mouse model [260,261], and in mice with Ang II-induced heart failure, which is a model for heart failure in elderly people [262]. These findings suggest that senolytics may be effective against some forms of heart failure, especially fibrotic subtypes. HFpEF, characterized by abnormal diastolic function due to stiffness of the left ventricle, is a common problem in elderly patients, for which an effective treatment has not yet been discovered. Senolytics could be a promising treatment in addition to other heart failure drugs such as ACE inhibitors, ARBs, and the newer sodium-glucose transport protein 2 (SGLT2) inhibitors.

In atrial fibrillation (AF), the most common arrhythmia worldwide affecting aged individuals, pathological fibrosis is theorized to disrupt electrical conductance through cardiomyocyte gap junctions in the atrium [263,264]. Senescent cell accumulation in the atrium has been observed in rodents and humans with AF and heart failure [265,266], and senolytic treatments have been shown to attenuate atrial senescent cell accumulation in rodents. There are other preclinical studies using quercetin or fisetin that suggest potential for alleviating atrial fibrosis and fibrillation in rodents, although these drugs were used as anti-fibrotic drugs and effects on senescent cells were not explored. Quercetin alleviated fibrosis in the atrium in isoproterenol (ISO)-induced tachycardia rat models, the suggested underlying mechanism being increased miR-135b expression inhibiting the TGF-β/SMAD pathway [267]. Fisetin improved left atrial fibrosis in an MI-induced AF rat model related to increased levels of phosphorylated AMPK (p-AMPK) and decreased NF-κB p65, p38MAPK, and smad3 phosphorylation [268]. However, AF is one of the diseases that has discordances between humans and rodents; rodent hearts do not develop AF naturally [268]. In the fisetin study, the median duration of AF measured in an ex vivo perfused rat heart was only 6.96 s in the non-fisetin treated control group [268]. More research is needed to test effects of senolytic treatments using larger animals that better reflect human arrythmias.

While fibrotic diseases are a promising target for senotherapies, there are a few reports that cellular senescence suppresses fibrosis. In the human atrium, *p16^INK4a^* expression and SA-β-gal are positively correlated with fibrotic lesions [269]. This finding was replicated in mice deficient in the senescence-driving kinase upstream of p53, ataxia-telangiectasia mutated (ATM) [187]. While fully formed senescent cells are profibrotic, senescent fibroblasts do not proliferate, and therefore in some circumstances, cellular senescence itself might also be anti-fibrotic. In many cases, pathological fibrosis is an adverse consequence in disease, but it is important to recognize that physiological fibrosis can be protective. For example, fibrosis after myocardial infarction replaces necrotic myocytes and maintains cardiac wall integrity, and the fibrous cap of atherosclerotic plaques prevents plaque rupture. Unlike antifibrotic drugs, senolytics and senomorphics only target dysfunctional senescent fibroblasts and do not appear to inhibit physiological fibrosis by non-senescent fibroblasts, and thus may be therapeutic candidates for fibrotic diseases, an area deserving of further study.

Ischemic heart disease, also known as coronary artery disease, causes myocardial necrosis leading to heart failure and other sequelae due to the low regenerative capacity of the adult heart. D+Q has been shown to activate approximately 10% of cardiac resident CPCs in aged mice [7] and in high-fat diet-induced heart failure model in mice [185]. In mouse and rat models of ischemia reperfusion, navitoclax appeared to alleviate myocardial fibrosis and hypertrophy [270,271] and improved left ventricular ejection fraction with increased survival in aged mice [54,272,273]. These findings suggest that senolytic treatment may be effective in restoring cardiac regenerative capacity by activating CPCs and increasing new cardiomyocyte and blood vessel formation. Accumulating evidence suggests that cellular therapy, in which several types of cells are injected, may alleviate cardiac disease [274,275]. Potentially, treating cells before they are transplanted with anti-senescence drugs may be useful for promoting direct cardiogenic differentiation due to removal of senescent CPCs.

Advances in treatments have improved survival rates in cancer patients, and side effects of cancer treatments, particularly in the cardiovascular system, have become an important issue for cancer survivors [276,277]. The field of cardio-oncology has received increasing attention in recent years. However, many aspects of both radiation-induced and cancer drug-induced CVDs have yet to be fully elucidated. Doxorubicin, used for treating leukemias as well as breast, gastric, lung, liver, and kidney cancers, stops DNA replication by inhibiting topoisomerase II. The most common side effect is cardiomyopathies. Doxorubicin is also known to cause cellular senescence by disrupting DNA replication. Therefore, senescent cell accumulation may be associated with these toxic side effects, as indicated in vivo by increased p16^INK4a^ positive CPCs being found in heart tissue biopsies from patients treated with doxorubicin and increased cardiac p53 expression in doxorubicin-treated mice [278]. Radiation therapy may also increase senescent cell burden due to off-target DNA damage. Senolytic therapies could alleviate side effects of cancer treatments, and possibly allow use of more aggressive anti-cancer treatments.

In atherosclerotic diseases, senescent ECs, VSMCs, fibroblasts, and inflammatory cells cause or worsen plaque buildup and destabilize existing plaques. Senolytics such as navitoclax [162,279], anti-GPNMB vaccination [222], digoxin [217,218,280], BPTES [220], and 17-DMAG (alvespimycin) [281], reduce atherosclerotic plaque formation and inflammation in atherosclerotic model mice. Fibrous capsule thinning, a risk factor for thrombosis, is decreased by senolytic administration in LDL receptor (Ldlr) knockout (KO) mice [130]. Senolytic treatment may reduce vascular calcification by removing senescent VSMCs [282], a major problem in coronary artery angioplasty and bypass surgery, and potentially may increase the success rates of these operations. A catheter-based procedure, percutaneous transluminal coronary angioplasty (PTCA), percutaneous coronary intervention (PCI), or endovascular therapy (EVT) can be used in sclerotic arteries to widen the narrowed or blocked artery and improve blood flow. A stent (a small, metal mesh tube) is placed inside the artery to help keep it open and prevent the artery from narrowing again. Potentially fatal restenosis can occur following the treatment due to neointimal hyperplasia, caused mainly by the excessive growth of VSMCs in the blood vessel wall. Interestingly, coating stents with navitoclax had a protective effect against restenosis in rabbits treated with a high-cholesterol diet [283]. D+Q has been shown to decrease senescent cell burden in arteriovenous fistulae allowing hemodialysis in chronic kidney disease model mice [284]. Aortic dissections and aneurysms are other conditions with high mortality. They are both tied to senescent cell accumulation, evidenced by high numbers of SA-ßgal, p53, and p21 ^CIP1/WAF1^ positive cells in patients with thoracic aortic aneurysms and dissections. Senescent VSMCs seem to cause the vessel wall to be weakened against hemodynamic pressure, contributing to aneurysm formation. D+Q reduces Ang II-induced abdominal aortic aneurysm (AAA) size in aged mice [285]. UBX1967, a BCL-xL small molecule inhibitor, appears to reduce ischemic retinopathy in an oxygen-induced mouse retinopathy model [286].

Pulmonary arterial hypertension (PAH) is caused by thickening of the muscle (medial) layer in pulmonary arteries and arterioles. PAH can lead to irreversible and detrimental cardiac changes as the right ventricle adapts to high pressure and medial thickening in the arteries also becomes irreversible over time. There is no cure for PAH so far; treatments can only alleviate symptoms, and in some cases, lung transplantation is necessary. A recent study has brought attention to senescent VSMC accumulation as a potential cause of irreversible PAH, and senolytic treatment successfully alleviated the condition in a monocrotaline- and shunt-induced PAH rat model [108]. However, anti-p53 senescent smooth muscle cell senolytic therapy did not attenuate declines in right ventricular function [135]. More evidence is needed. It is difficult to develop accurate PAH animal models due to anatomic differences between rodents and humans.

Peripheral artery disease (PAD), also known as vascular claudication, is caused by narrowing of the arterial lumen due to atherosclerotic plaque buildup in the extremities. Critical limb ischemia is a serious consequence of PAD in which there is inadequate blood flow to the extremities, causing ischemic tissue damage that can eventually necessitate amputation in 10 to 40% of cases and may even cause death from necrosis and infection. In addition to atherosclerosis, reduced angiogenetic capacity to allow bypassing the narrowing vessels due to EC dysfunction and senescence may be an important contributor. In a murine model of induced limb ischemia, old mice exhibited increased senescence markers, such as p16^Ink4a^ and p21^Cip1/Waf1^, in the ischemic limb along with reductions in factors such as VEGF-A, HIF1α, and PPARγ coactivator 1α. Targeting EC senescence using *EC-p53 KO* mice improved vasodilation and angiogenesis [95,96]. Advances in regenerative medicine have enabled the development of cell-based therapies that promote the formation of new blood vessels, “therapeutic angiogenesis” [287,288,289]. As mentioned, senolytic treatment may enhance these cell transplant strategies by reducing senescent progenitor cell burden prior to transplantation. Heart transplantation is a treatment option for severe heart disease. In hearts to be transplanted, D + Q reduces senescent cell burden, alleviates age-associated inflammation, and prolongs survival of cardiac allografts from old mice [261], suggesting that senolytics may be useful for increasing transplanted organ viability and reducing transplant rejection.

Systolic hypertension, characterized by high systolic blood pressure and normal diastolic blood pressure, is a common problem in the elderly [290]. It is associated with a two- to fourfold increase in the risk of myocardial infarctions, left ventricular hypertrophy, kidney dysfunction, stroke, and cardiovascular mortality [291,292]. D+Q may alleviate hypertension in the elderly as it improves vasorelaxation and vasomotor function in aged mice [47,282]. Cellular senescence caused by macromolecular damage, inflammation, deregulated nutrient sensing, and telomere modification is associated with systolic hypertension [293,294]. D+Q improved both endothelial and vascular smooth muscle cell dependent vasorelaxation in aged mice [47,282].

The aging lung is prone to low-grade chronic inflammation, with inflammatory SASP factors such as IL-6 being present at greater abundance in bronchoalveolar lavages from elderly compared to young patients. Factors such as cigarette smoke exposure and other inhaled toxins can drive senescence and increase release of SASP factors. Senescent cells can express many inflammatory and pro-fibrotic factors, such as MMPs, that disrupt tissues and the extracellular matrix and induce replacement of functioning organ tissue by fibrotic tissue. Senescent cell accumulation increases susceptibility to infections, disrupts alveolar gas exchange, and impairs repair after pulmonary injury. Senescent pulmonary cells secrete SASP factors that can accelerate malignancies [295,296,297]. Fibrotic pulmonary diseases such as COPD and IPF have been linked to senescent cell accumulation [23,108,217,298,299].

Finally, preeclampsia, a hypertensive disorder that can occur during pregnancy, has been shown to be associated with pathological senescent endothelial cell and syncytiotrophoblast accumulation [300]. Increased senescent cell burden and high local SASP factors in the uterus have also been connected to preterm birth [301]. Senolytics may improve the health of mothers and newborns; however, whether senolytics would be safe during pregnancy is highly questionable.

**Table 3:** Cardiovascular diseases alleviated by senolytics in preclinical models.

Model Type	Senescent Cell Removal Model	Senolytic Drug	Result of Senolytic Therapy	Reference
Aging (mouse)		D+Q	Improved left ventricular ejection fraction	[47]
	*INK-ATTAC*	D+Q	Activated resident cardiac progenitor cells and cardiomyocyte formation	[7]
	*INK-ATTAC*	Navitoclax	Alleviated myocardial hypertrophy and fibrosis	[54]
		D+Q	Prolonged survival of old cardiac allografts	[261]
		D+Q	Improved vascular relaxation	[47]
	*INK-ATTAC*	D+Q	Reduced aortic calcification and improved vasomotor function	[282]
High-fat diet-induced heart failure (rat)		Q	Attenuated high-fat diet-induced cardiac systolic dysfunction	[259]
High-fat diet-induced heart failure (mouse)		Q	Normalized heart size and reduced cardiac fat and fibrosis	[8]
High-fat diet-induced heart failure (mouse)		D+Q	Improved CPC proliferation and cardiac repair	[185]
Angiotensin II (Ang II)-induced heart failure (mouse)		Navitoclax	Improved left ventricular ejection fraction	[262]
Doxorubicin (DOX)-induced heart failure (mouse)		Navitoclax	Improved cardiac function	[260]
Ischemia–reperfusion injury (mouse)		Navitoclax	Alleviated myocardial hypertrophy and fibrosis, improved left ventricular ejection fraction, increased myocardial vascularization	[272]
Aged mice of following MI (mouse)		Navitoclax	Increased survival after myocardial infarction	[273]
Ischemia and reperfusion-injury (rat)		Navitoclax	Improved cardiac function and increased angiogenesis	[270]
Myocardial infarction (mouse)		D+Q	Improved cardiac function, increased regeneration	[271]
AF after acute myocardial infarction (rat)		Fisetin	Attenuated atrial fibrosis and AF duration following acute myocardial infarction	[268]
Isoproterenol induced AF (rat)		Q	Alleviated fibrosis and collagen deposition in atrial tissues	[267]
Atherosclerosis (Ldlr KO) (mouse)	*INK-ATTAC* *p16-3MR*	Navitoclax	Reduced atherosclerotic plaque formation and instability	[162]
	*INK-ATTAC* *p16-3MR*	Navitoclax	Improved fibrous cap thickness	[130]
Atherosclerosis (ApoE KO) (mouse)	*p16-3MR*	Navitoclax	Reduced atherosclerotic plaque size and inflammatory cell count	[279]
		GPNMB vaccine	Reduced atherosclerotic plaque formation and inflammation	[223]
		Digoxin	Reduced atherosclerotic lesion formation and plasma lipid levels	[280]
		17-DMAG	Reduced atherosclerotic lesions and induced a more stable plaque phenotype	[281]
		BPTES	Reduce atherosclerotic plaque formation	[220]
Dialysis arteriovenous fistula (mouse)		D+Q	Decreased senescent cells in dialysis arteriovenous fistula mice	[284]
MCT and shunt-induced PAH (rat)		Navitoclax	Improved hemodynamic and structural changes associated with severe PAH	[108]
Oxygen-induced retinopathy (mouse), eye tissue (human)	*INK-ATTAC*	UBX1967	Ameliorated ischemic retinopathy	[286]
Angiotensin II (Ang II) administrated, aged (mouse)		D+Q	Reduced AAA size	[285]
Cholesterol diet (rabbit)		Navitoclax	Reduced stenosis area of senolytic-coated stent	[25]

## 7. Conclusions

Increasing senescent cell burden in the cardiovascular system with aging parallels the increasing prevalence of cardiovascular diseases in elderly patients. Senescent cell accumulation can be detected in virtually every cardiovascular tissue and organ. Anti-senescent cell therapies, along with other strategies targeting the fundamental aging mechanisms, have potential to increase the healthy years of our lives. Besides pharmaceutical interventions, lifestyle modifications such as caloric restriction and exercise may enhance healthspan due to multifactorial effects, including but not limited to reduced senescent cell burden. According to The Unitary Theory of Fundamental Aging Mechanisms that builds on the Geroscience Hypothesis, senotherapies could have effects like those of these interventions because their effects on fundamental aging processes may interact. Cardiovascular diseases lead to high morbidity and mortality burden and are a primary target for strategies targeting fundamental aging mechanisms. Challenges include lack of long-term clinical studies evaluating the effects of systemic senescent cell elimination. Even in the case of senomorphics, while some drugs such as metformin appear to be well studied and safe, long-term effects on systemic and local tissue homeostasis are not fully understood. For example, some senomorphic compounds might be immunosuppressive. Cell type-specific senolytics might provide information about different forms of senescent cells and their contribution to cardiovascular diseases. Several dozen new senolytic agents have been discovered and are currently being tested extensively. When targeting senescent cells in vivo, it is important to recognize that cellular senescence is a mechanism that prevents tumorigenesis and is important during embryogenesis, possibly wound healing (although removing senescent cells appears to enhance healing of chronic wounds [302,303], and host immunity. The distinction between physiological, beneficial senescence and pathological senescence is crucial, especially the impact of persistent senescent cells that have evaded removal by the immune system. For example, inhibition of senescent cell formation by suppressing p53 and other proteins can increase risk for malignancies so the prevention of formation of senescent cells may be detrimental, while removing persisting senescent cells appears to decrease tumorigenesis and cancer spread [304,305,306]. It is important to proceed with clinical trials carefully, taking these points into consideration. Local delivery methods that can act on specific senescent cardiovascular cells or tissues, along with further studies to optimize the dosage, mode of administration, and drug combinations, may enable optimization of anti-senescence treatment regimens. Since these therapies targeting the underlying etiology of aging have also been shown to have effects on risk factors such as hypertension and diabetes, if promising data are obtained, these therapies could be used as monotherapy for cardiovascular diseases and prevent polypharmacy, the use of multiple medications, which is one of the problems of elderly care. They may offer an alternative to patients who, due to very advanced biological age, cannot tolerate established drugs. To evaluate the effect of these novel agents, clinical gerodiagnostic composite scores are needed. There are currently no highly sensitive and specific individual markers for senescent cardiovascular cells in vivo [68]. This is mainly due to the heterogeneity of senescent cells. With accumulating evidence in preclinical studies, some therapies targeting senescent cells have progressed to the point of early clinical trials. Studies using animals other than rodents, such as rabbits, dogs, pigs, and monkeys, may provide much insight into the effect of targeting senescence in the cardiovascular system and could facilitate translation to patients. While the field is still new and needs much refinement, targeting senescent cells could transform cardiovascular disease treatment and markedly improve patient quality of life, not to mention potential systemic effects against a range of diseases beyond those of the cardiovascular system.

## Figures and Tables

**Table 1:** Possible cell-type specific senescence markers for classifying senescent cells in the cardiovascular system. The ↑ and ↓ symbols indicate increases or decreases, respectively, in protein synthesis or activity.

Cell Type	SASP Factors	Possible Cell-Type Specific Senescence Markers and Hallmarks
Endothelial cells	IL-1, IL-6, IL-8, Il-15, MCP-1, TNF-α, SELP, ANGPTL-2, HTRA4, PCNA, CHEK4, and VEGF	Down-regulation of nitric oxide (NO) and eNOS
		Prothrombotic metalloprotease (PAI-1, and PAI-2) activity
		Expression of adhesion molecules (ICAM-1, VCAM, and PECAM-1)
		Production of Angiotensin II (Ang II) and Endothelin 1
		IGF binding protein expression, such as IGFBP-5
		microRNA34a
Vascular smooth muscle cells	IL-1a, IL-6, IL-8, TGF-ß1, and MMPs, CCL-3/CCL-4, and MCP-1	Calcification markers (OPN, OPG, Runx-2, BMPs, and ALP) ↑
		Fibrosis markers (elastase ↑, collagen ↓)
		Muscle contraction markers (α-SMA ↓, SM-MHC ↓and calponin ↓)
		microRNA34a
Cardiomyocytes	IL-6, EDN3, TGF-ß2 and GDF15	Fatty acid oxidation enzyme (CPT1) activity ↓
		Glutathione reductase activity ↓
		PPARα and PGC-1α activity ↓
		Troponin I phosphorylation ↓
Macrophages	IL-1ß, IL-6, TNF-α, and MMP-3, and MMP-13	TM1, SIPR1, TAM, MERTK and BRD4
T cells	IL-6, TNF-α, IFN- γ and OPN	Perforins and granzymes ↑
		Down-regulation of CD27 and CD28
		CD4^+^ CD44^high^ CD62L^low^ PD-1^+^ CD153^+^
Fibroblasts		Myofibroblast markers (POSTN, PARP1, IL-6, IL-13, THBS1, and TGF-ß1)
		Matricellular protein (CCN1) ↑

**Table 2:** Senescence initiators, pathological processes, and associated cardiovascular diseases.

**Cell Type**	**Initiators**	**Pathology**	**Diseases**
Endothelial cells	Telomere shortening Shear stress from blood flow Dyslipidemia AngII signaling LDL oxidation Hyperinsulinemia Doxorubicin Irradiation ROS	Local and systemic inflammation Impaired vascular relaxation Hindered blood flow regulation Hypercoagulability Angiogenesis ↓ Expression of adhesion molecules ↑	Hypertension Atherosclerosis Peripheral artery disease Ischemic heart disease
Vascular smooth muscle cells	Telomeric dysfunction AngII signaling Dyslipidemia Hyperinsulinemia ROS	Vascular calcification Fibrosis Impaired smooth muscle contraction Hindered blood flow regulation Atherosclerotic plaque vulnerability	Hypertension Aortic aneurysm Aortic dissection Atherosclerosis Myocardial infarction
Cardiomyocytes	Pressure overload Hypoxia Cardiotoxic drugs Irradiation ROS	Cardiac dysfunction Cardiac hypertrophy Fibrosis	Heart failure Cardiomyopathy Ischemic heart disease Arrhythmias
Immune cells	Dyslipidemia Cell debris ROS	Inflammation Impaired cardiac electrical conduction	Atherosclerosis Heart failure Arrhythmias
Fibroblasts	Pressure overload Hypoxia Cardiotoxic drugs Irradiation ROS	Fibrosis Cardiac dysfunction Cardiac hypertrophy	Heart failure Cardiomyopathy Arrhythmias

## Data Availability

Not applicable.

## Abbreviations

53BP1	Tumor suppressor p53-binding protein 1
ACE	Angiotensin-converting enzyme
ARBs	Angiotensin receptor blockers
AFAR	American Federation for Aging Research
AKT1	Threonine protein kinase
ALP	Alkaline phosphatase
AMPK	AMP-activated protein kinase
Ang II	Angiotensin II
ANGPTL2	Angiopoietin-like protein 2
ApoE	Apolipoprotein E
BCL-xL	B-cell lymphoma-extra large
BMP-2	Bone morphogenetic protein 2
BPTES	Bis-2-(5-phenyl- acetamido-1,3,4-thiadiazol-2-yl) ethyl sulfide
CABG	Coronary artery bypass graft
CAR-T	Chimeric antigen receptor T cell
CCL3	Chemokine (C-C motif) ligand 3
CCN1	CCN family member 1
CCR2	C-C motif chemokine receptor 2
CF	Cardiac fibroblasts
CMV	Cytomegalovirus
COPD	Chronic obstructive pulmonary disease
CPC	Cardiac progenitor cell
CPT1	Carnitine palmitoyltransferase I
CVD	Cardiovascular disease
D	Dasatinib
DAMP	Damage-associated molecular pattern
DNA	Deoxyribonucleic acid
DNA-SCARS	DNA segments with chromatin alterations reinforcing senescence
EC	Endothelial cell
ECM	Extracellular matrix
Edn3	Endothelin 3
EFNB1	Ephrin B1
eNOS	Endothelial nitric oxide synthase
EPCs	Endothelial progenitor cells
ET-1	Endothelin 1
FAS	TNF receptor superfamily member 6
FGF	Fibroblast growth factor
FOXO	Forkhead box protein O
GDF15	Growth differentiation factor 15
GH	Growth hormone
GHRH	Growth hormone–releasing hormone
GLS1	Glutaminase 1
GPNMB	Glycoprotein nonmetastatic melanoma protein B
HF	Heart failure
HFpEF	Heart failure with preserved ejection fraction
HGPS	Hutchinson–Gilford progeria syndrome
HIF	Hypoxia-inducible factor
HMGB-1	High mobility group box 1
hs-CRP	High sensitivity C-reactive protein
HSP	Heat shock protein
HSP90	Heat shock protein 90
ICAM-1	Intercellular adhesion molecule-1
ICD	International Classification of Diseases
IFN- γ	Interferon-gamma
IGF-1	Insulin-like growth factor 1
IGFBP	Insulin-like Growth Factor-binding Protein
IL	Interleukin
KGA	Kidney-type glutaminase
Ki-67	Marker of proliferation Ki-67
KO	Knockout
LDL	Low-density lipoprotein
MAPK	Mitogen-activated protein kinase
MCP-1	Monocyte chemoattractant protein-1
MI	Myocardial infarction
miRNAs	MicroRNAs
MMP	Matrix Metallopeptidase
mTOR	Mammalian target of rapamycin
NAD+	Nicotinamide adenine dinucleotide
NADPH	Nicotinamide dinucleotide phosphate
NAFLD	Non-alcoholic fatty liver disease
NK	Natural killer
NO	Nitric oxide
NOXs	Nicotinamide dinucleotide phosphate oxidases
OIS	Oncogene induced senescence
OPG	Osteoprotegerin
OPN	Osteopontin
OSKM	The “Yamanaka factors”, Oct4, Sox2, Klf4, and c-Myc
p16Ink4a	Cyclin-dependent kinase inhibitor 2A
p21^CIP1/WAF1^	Cyclin-dependent kinase inhibitor 1 (Cdkn1a)
p38MAPK	p38 mitogen-activated protein kinase
p53	Cellular tumor antigen p53
PAH	Pulmonary arterial hypertension
PAI-1	Plasminogen activator inhibitor-1
PDGF	Platelet-derived growth factor
PI3K	Phosphoinositide 3-kinases
PPARα	Peroxisome proliferator-activated receptor α
Q	Quercetin
RAAS	Renin-angiotensin-aldosterone system
RNA	Ribonucleic acid
ROS	Reactive oxygen species
Runx-2	runt-associated transcription factor 2
SADs	Senescence-associated distention of satellites
SAHF	Senescence-associated heterochromatin foci
SASP	Senescence-associated secretory phenotype
SA-βgal	Senescence-associated beta-galactosidase
SCAPs	Senescent cell anti-apoptotic pathways
scRNA-seq	Single cell RNA-sequencing
SGLT2	Sodium-Glucose Transport Protein 2
SIRT	Sirtuin
SM-MHC	Smooth muscle myosin heavy chain
SOD	Superoxide dismutase
TAFs	Telomere-associated foci
TAME	Targeting Aging with Metformin
TEM	Transmission electron microscopy
TGFβ	Tumor growth factor-beta
TIS	Therapy-induced senescent cells
TNFR1	TNF receptor 1
TNF-α	Tumor necrosis factor alpha
uPAR	Urokinase-type plasminogen activator receptor
VEGF	Vascular endothelial growth factor
VSMC	Vascular smooth muscle cell
X-Gal	X-galactosidase
α-SMA	α-smooth muscle actin
γ-H2AX	Phosphorylated H2A histone family member X
